# Seed Biopriming with Salt-Tolerant Endophytic *Pseudomonas geniculata*-Modulated Biochemical Responses Provide Ecological Fitness in Maize (*Zea mays* L.) Grown in Saline Sodic Soil

**DOI:** 10.3390/ijerph17010253

**Published:** 2019-12-30

**Authors:** Shailendra Singh, Udai B. Singh, Mala Trivedi, Pramod Kumar Sahu, Surinder Paul, Diby Paul, Anil Kumar Saxena

**Affiliations:** 1 Plant-Microbe Interaction and Rhizosphere Biology Lab, ICAR-National Bureau of Agriculturally Important Microorganisms, Kushmaur, MaunathBhanjan 275 103, India; singh.shailendra512@gmail.com (S.S.); pramod15589@gmail.com (P.K.S.); surinderpaulsandhu@gmail.com (S.P.); saxena461@yahoo.com (A.K.S.); 2 Amity Institute of Biotechnology, Amity University Uttar Pradesh, Lucknow 227 105, India; mtrivedi@lko.amity.edu; 3 Pilgram Marpeck School of Science, Technology, Engineering and Mathematics, TruettMcConnel University, 100 Alumni Dr., Cleveland, GA 30528, USA

**Keywords:** *Pseudomonas geniculata*, seed biopriming, ecological fitness, maize, soil salinity and sodicity, salt stress

## Abstract

Under changing climate, soil salinity and sodicity is a limiting factor to crop production and are considered a threat to sustainability in agriculture. A number of attempts are being made to develop microbe-based technologies for alleviation of toxic effects of salts. However, the mechanisms of salt tolerance in agriculturally important crops are not fully understood and still require in-depth study in the backdrop of emerging concepts in biological systems. The present investigation was aimed to decipher the microbe-mediated mechanisms of salt tolerance in maize. Endophytic *Pseudomonas geniculate* MF-84 was isolated from maize rhizosphere and tagged with green fluorescent protein for localization in the plant system. Confocal microphotographs clearly indicate that MF-84 was localized in the epidermal cells, cortical tissues, endodermis and vascular bundles including proto-xylem, meta-xylem, phloem and bundle sheath. The role of *P. geniculate* MF-84 in induction and bioaccumulation of soluble sugar, proline and natural antioxidants enzymes in maize plant was investigated which lead not only to growth promotion but also provide protection from salt stress in maize. Results suggested that application of *P. geniculate* MF-84 reduces the uptake of Na^+^ and increases uptake of K^+^ and Ca^2+^ in maize roots indicative of the role of MF-84 in maintaining ionic balance/homeostasis in the plant roots under higher salt conditions. It not only helps in alleviation of toxic effects of salt but also increases plant growth along with reduction in crop losses due to salinity and sodicity.

## 1. Introduction

Soil salinization, environmental pollution, water scarcity and growing world population are considered greater threats to global food production and supply in the 21st century. As the global population keeps on increasing day by day, the simultaneous reduction in the available land for crop cultivation is worsening the situation and doubling the challenges for researchers and policy makers [1,2,3]. Various environmental stresses such as soil salinity; extreme temperatures, drought and flood have affected the crop cultivation and agricultural production. Among these, increased salt concentration in soil (salinity and sodicity) is one of the most important environmental stresses [3]. It causes substantial reduction in cultivable land area ultimately leading to reduced crop production [3,4]. It has been estimated that worldwide 20% of total cultivated and 33% of irrigated agricultural lands are afflicted by high salinity. Furthermore, the salinized areas are increasing at a rate of 10% annually owing to various factors, including low precipitation, high surface evaporation, weathering of native rocks, irrigation with saline water and poor cultural practices. It has been estimated that more than 50% of the arable land would be salinized by the year 2050 [3]. Salt stress affects plant growth and yield in many crop species including cereals (wheat, rice and maize), forages (clover), pulse crops (pea, chickpea, pigeonpea, etc.) and horticultural crops (potato and tomato) [3]. However, maize is relatively more susceptible to excessive salt concentration [1,2].

Globally, maize is the third most important crop after rice and wheat. Over last six decades, in India, maize production has increased from 1.73 million metric tonnes (mt) during 1950–1951 to as high as 26.88 mt during 2016–2017 which demonstrates the strides made in maize research. Under changing climate, both biotic and abiotic stresses cause significant losses in maize production. Among abiotic stresses, soil salinization is the major cause of low productivity in maize [1,2]. It is one of the most important environmental factors limiting the crop productivity as maize plants are moderately sensitive to salinity. Ions that contribute to sodicity/salinity in the soil include Cl^−^, SO_4_^2−^, HCO^3−^, Na^+^, Ca^2+^, Mg^2+^, and rarely, NO^3−^ or K^+^ [1,2,3]. Increasing human population and reduction in arable land are two major threats to the agricultural sustainability [4]. The rate of reduction in arable land has increased in the recent decades which is attributed not only to the corresponding increase in urbanization but also increase in the rate of salinization of arable land. In the saline sodic soil, high osmotic stress, nutritional imbalance and toxicities, poor soil physical and biochemical conditions importing poor crop establishment lead to reduced crop productivity [5]. High salt concentrations in soil interfere with the uptake of some essential elements leading to nutritional imbalance in the plants grown in such soils [6]. Excessive accumulation of Na^+^ in cell walls causes rapid osmotic burst and programmed cell death [7]. Further, significantly higher accumulation of some elements, *viz*. Na, Cl and B in the cytosol has specific toxic effects on plants [3,5,7].

Several strategies have been developed and adopted in order to alleviate the toxic effects caused by salt stress in plants including rhizosphere engineering and plant genetic engineering [8,9]. It is being attempted through broadening the genetic base of breeding material, improved agronomic practices and reclamation of salt affected soils although these methods are costly and time consuming [9,10,11]. Use of salt-tolerant cultivars (hybrid and non-hybrid) is an important approach conferring sustainability to the crop production but the availability of salt-tolerant gene(s)/effective donor parent has largely remained unattainable and it seems to be the major limitation of this approach [9,10,11]. It has obligated researchers to explore alternate strategies to reduce the negative effects of salt stress in economically important crop plants [9]. Salt-tolerant rhizospheric and endophytic microorganisms have emerged as a promising supplement in the approaches to crop protection [1,12,13,14,15].

In plant-microbe interactions, the beneficial microorganisms colonize and proliferate in the rhizosphere/endorhizosphere of crop plants and elicit plant growth through various direct and indirect mechanisms [1,16,17]. Recent studies suggest that application of plant growth promoting rhizobacteria (PGPR) and endophytes have become a proven alternative to alleviate stress caused by higher salt concentrations [1,18,19]. However, exploration of the role of endophytes in the management of biotic and abiotic stresses is gaining importance. Systemic (true) and non-systemic (transient) endophytes establish a close relationship with plants often being engaged in mutualism with the latter. However, endophyte-plant relationships have not yet been well understood [3,13,20,21]. Endophytes appear to enhance the growth of their plant symbionts by increasing the uptake of land limited nutrients from the soil such as phosphorus, zinc, copper, boron and making other nutrients available to plants such as rock phosphate and atmospheric nitrogen which are normally trapped informs inaccessible to plants [3,13,22]. Recent studies have shown that endophytic microorganisms elicit induced systemic tolerance (IST) by induction of physiological and biochemical changes in the plants which lead to enhanced tolerance to salt stress [23,24,25]. The endophytic bacteria facilitate plant growth indirectly by reducing negative impact of salt stress by production and regulation of the phytohormones (e.g., cytokinin, auxin, and gibberellins), antioxidant enzymes, lowering of plant ethylene levels and/or by uptake and translocation of Na^+^ in the plants [26,27,28]. Interactions among microorganisms, roots, soil and water in the rhizosphere are complex and dynamic and may induce changes in physico-chemical and structural properties of the soil [5,29]. Moreover, polysaccharides produced by bacteria bind soil particles to form macro- and micro-aggregates. After some time, pores formed between micro-aggregates are used by fine roots and fungal hyphae stabilizing macro-aggregates. Plants treated with the exo-polysaccharides (EPS) and/or EPS producing bacteria showed tolerance to salt stress due to improved soil structure [5,19,29]. Further, EPS can also bind to cations including Na^+^ and thus, making it unavailable to plants under salt stress conditions [1,29]. Considering the importance of the subject, this study was specifically designed to study the impact of seed bio-priming with salt-tolerant endophytic *Pseudomonas geniculata* which modulates the biochemical responses and provides ecological fitness to the maize (*Zea mays* L.) grown in saline sodic soil.

## 2. Materials and Methods

### 2.1. Characterization and Identification of Microbial Strains

During the course of our investigation, maize rhizosphere soil and root samples were collected from different parts of India. A total of 450 morphotypes of bacteria were isolated on HiChrome Bacillus Agar medium (HiMedia, Mumbai, India). These morphotypes were further screened and characterized for salt tolerance [9]. The antimicrobial activities was evaluated following the methods of Singh et al. [30], Briefly, test strains were placed at the edge of PDA plate, and mycelial plug (5 mm) of phytopathogenic fungi *Rhizoctoniasolani* was inoculated at the centre of the same PDA plate. Thereafter, plates were sealed with Parafilm (Tarson, Kolkata, India) strips and incubated for 5 days at 26 ± 2 °C. For each test strain, there were five replicate (plates) and the experiment was repeated twice. Finally, percent inhibition of mycelial growth was calculated. The plant growth promoting traits, such as solubilization of phosphate, potash and zinc, IAA production, siderophore production of the test strains were evaluated under *in vitro* conditions as per the methods described by Singh et al. [30]. Briefly, phosphate solubilization ability was evaluated on NBRI-P medium. However, Potash solubilization efficiency of the bacterial strains was tested on Alekzendrov medium containing mica and feldspar as the source of mineral potash. The selected bacterial strains were screened for their zinc solubilizing ability. For this, five insoluble zinc compounds *viz*. zinc sulfate (ZnSO_4_), zinc oxide (ZnO), zinc chloride (ZnCl_2_), zinc phosphate Zn_3_ (PO_4_)_2_ and zinc carbonate (ZnCO_3_) were taken. The selected bacterial strains were spotted aseptically on the respective zinc medium plates and incubated for 7 days at 28 °C. Zinc solubilizing strains produced clear zones around colonies on the plates. The potential isolates having salt tolerance and plant growth promoting traits were separated from the group. From the group, the most potential isolates MF-30 and MF-84 were identified using 16S rRNA gene sequence similarity. For this, bacterial DNA was isolated using Wizard^®^ Genomic DNA Purification Kit (Promega, Madison, WI, USA). Further, 16S rRNA gene amplification was done using universal primers set *viz*. forward primer: AGAGTTTGATCCTGGCTCAG; reverse primer: AAGGAGGTGATCCAGCCGCA [31]. The amplified product was sequenced by Eurofin Pvt. Ltd. (Bengaluru, India) and finally sequences were blast on EzBioCloud Database to identify them up to the species level. The phylogenetic tree was constructed using MEGA 5.0 software (Mega Limited, Auckland, New Zealand).

### 2.2. Root Colonization Assay

The maize seeds were bio-primed with MF-84 and sown in pots containing sterile soil mixture (sand:soil:vermiculite in 1:1:1 ratio). After 15 days of sowing, plants were uprooted gently, washed in running tap water and microscopy was done to see the colonization under Scanning Electron Microscope (S-3400N, Hitachi, Lawa, USA) as described by Singh et al. [32]. Briefly, root samples were fixed in osmiumtetraoxide solution (HiMedia) and 2.5% glutaraldehyde (HiMedia); dehydrated using gradient of ethyl alcohol (5%, 10%, 20%,50%, 70%, 90%, 100%) and dried under vacuum. Further, gold coating (20 nm) was done before visualization. Confocal microscopy was done at 488 and 543 nm laser lines using propidium iodide stain under Confocal Scanning Laser Microscope (Eclipse Confocal A1, Nikon, Kumagaya, Japan) as described by Singh et al. [32]. Confocal microscopic images indicated the endophytic nature of MF-84 and to confirm it Green Fluorescent Protein (GFP)-tagging was done.

### 2.3. Green Fluorescent Protein (GFP)-Tagging of the Pseudomonas Geniculata

The selected strain *P. geniculata* MF-84 used in the present study was tagged with Green fluorescent protein (GFP) and visualized under a confocal scanning laser microscope (Nikon). The mini-Tn5 gusA::gfp cassette was inserted into *Pseudomonas* spp. (recipient) by tri-parental mating with Tn5 gusA::gfp cassette (pFAJ1820) containing *E. coli* strain (donor) and pRK2013 plasmid harbouring *E. coli* HB101 (helper). The trans-conjugants were selected on a half-strength LB agar medium supplemented with kanamycin and nalidixic acid. The trans-conjugant colonies showing fluorescence under confocal microscope were picked up. The presence of GFP in the selected trans-conjugants was also confirmed by PCR amplification using the primers, *viz*. YL065 (F) 5′ GCGATGTTAATGGGCAAAAA-3′ and YL066 (R) 5′-TCCATGCCATGTGTAATCCT-3′. The PCR program for the amplification of desired 650-bp amplicon consisted of initial denaturation at 94 °C for 3 min, followed by 35 cycles at 94 °C for 30 s, annealing at 56 °C for 1 min, and extension at 72 °C for 1 min with the final extension at 72 °C for 10 min. The amplification was confirmed by agarose gel electrophoresis. The selected GFP-tagged trans-conjugants exhibiting morphological and growth rate characteristics similar to the wild type were used for further experiments.

### 2.4. In Planta Assay

#### 2.4.1. Soil Preparation and Analysis

Saline sodic soil was collected from Indian Institute of Seed Sciences agricultural farm, Kushmaur, Maunath Bhanjan, India. However, non-saline soil was collected from a farmer’s field, Mardah, Uttar Pradesh, India. Collected soil was sieved (2 mm pore size), air-dried and mixed with farm yard manure (3:1 *w/w*) and recommended doses of chemical fertilizers. Thereafter, soil was sterilized by autoclaving (121 °C for 30 min) twice at an interval of 24 h. The sterilized soil was kept static for five days at ambient room temperature. The soil properties were analyzed using standard protocols and procedures in triplicates and presented in Table 1.

#### 2.4.2. Experimental Set up

Maize seeds (*cv.* Sachin 777) were procured from an open market (Maunath Bhanjan, Uttar Pradesh, India). Seeds were surface sterilized with sodium hypochlorite (1% NaOCl) followed by three successive washings with sterile distilled water under aseptic conditions. Further, talc-based formulation of *P. geniculate* MF-84 was developed as per the methods given by Singh et al. [30] and colony forming unit of the end product was 2.57 × 10^8^. Maize seeds were bio-primed with talc-based formulation of *P. geniculate* MF-84 as per methods described by Singh et al. [9]. Seeds treated with sterile talc powder served as control. The experimental design comprised of six different treatments in three replications as given in Table 2. The experiments were set up as per treatments in a randomized block design under nethouse conditions. Each pot containing 5 kg of experimental soil was planted with 3 seeds under nethouse conditions. The experiments were carried out during July to October with relative humidity range between 80% and 90% under11/–13 h light/dark photoperiod. To maintain moisture content at field capacity (60%), pots were sprinkled with sterilized water every alternate day.

### 2.5. Sampling and Analysis

Maize leaves were sampled at 30 days after sowing (DAS) under nethouse experiments. Leaf samples were brought to the laboratory and total chlorophyll and total carotenoids content were measured following the methods as described by Sadasivam and Manickam [33]. However, total soluble sugar and proline content in the plant leaves were analyzed using the estimation protocols described by Thimmaiah [34]. Further, the effect of seed bio-priming was evaluated in terms of changes in the accumulation and activity of antioxidant enzymes in the plant roots after 30 DAS. The catalase activity [34]; peroxidase activity [33], and superoxide dismutase activity was measured using standard methods [35] with slight modifications [34]. Five plants were sampled randomly from each treatment to observe shoot and root length, fresh and dry weight of shoot and root at 30 DAS. The changes in the uptake of Na^+^, K^+^ and Ca^2+^ ions in maize roots were measured using a flame photometer at 30 DAS.

### 2.6. Statistical Analyses

The controlled laboratory experiments were carried out in completely randomized design (CRD) with five replications. However, nethouse experiments were laid out in randomized block design (RBD) with five replications. Data were subjected to analysis of variance and least significant difference (LSD) at *p* < 0.05 using statistical package for Social Sciences Version 16.0 program (SPSS Inc., Chicago, IL, USA, 2007). Data were compared with Duncan’s multiple range test at *p* < 0.05. Graphs were prepared using Microsoft Office Excel (2010).

## 3. Results

### 3.1. Characterization and Identification of Microbial Strains

**Figure 1:**
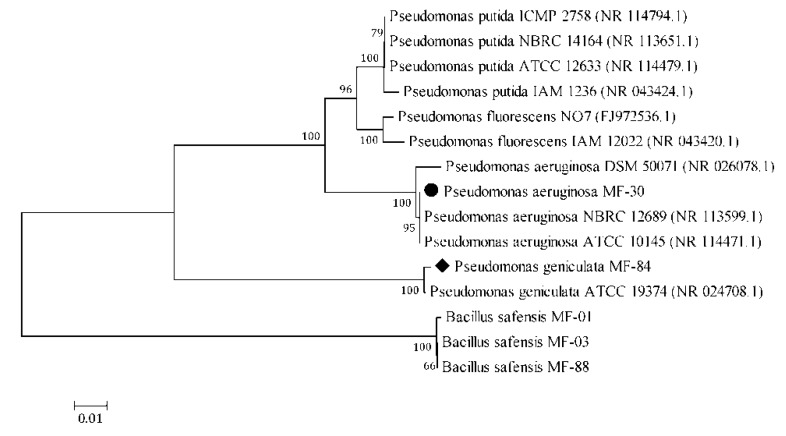
Neighbour joining tree derived by CLUSTAL W and MEGA 5.0 using analysis of 16S rRNA sequences of *Pseudomonas aeruginosa* MF-30 and *Pseudomonas geniculata* MF-84. The numbers at nodes indicate bootstrap support values, as calculated by MEGA 5.0.

Based on the 16S rRNA gene sequence similarity, strain MF-30 was identified as *Pseudomonas aeruginosa* (NCBI GenBank accession no. MH177243), while, MF-84 was identified as *Pseudomonas geniculata* (NCBI GenBank accession no. MK120890) (Figure 1). Results showed that *P. aeruginosa* MF-30 could tolerate and grow at 4.25% salt (NaCl) concentration. However, *P. geniculata* MF-84 could tolerate and grow at 5.5% salt (NaCl) concentration and hence selected for further *in planta* assay. It was observed that gradual increment in salt concentration reduces growth of the particular strain on the nutrient agar media in a proportional manner. Further, both the strains were found positive for phosphate, potash and zinc solubilization and IAA production under in vitro assay. *P. aeruginosa* MF-30 was found to produce siderophores, while *P. geniculata* MF-84 could not produce siderophores on chrome azurol S(CAS) medium.

### 3.2. Root Colonization Assay

**Figure 2:**
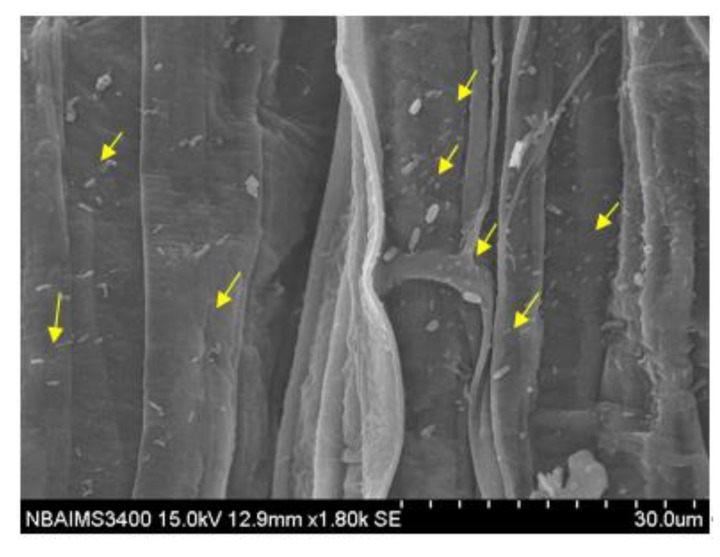
Scanning electron microphotographs showing root colonization of maize by *Pseudomonas geniculata* MF-84.

To investigate whether *P. geniculata* MF-84 was having ability to colonize maize root or not, an experiment was conducted under nethouse conditions. Scanning electron microphotographs clearly showed that 70% of the roots were colonized by *P. geniculata* MF-84 (Figure 2).

**Figure 3:**
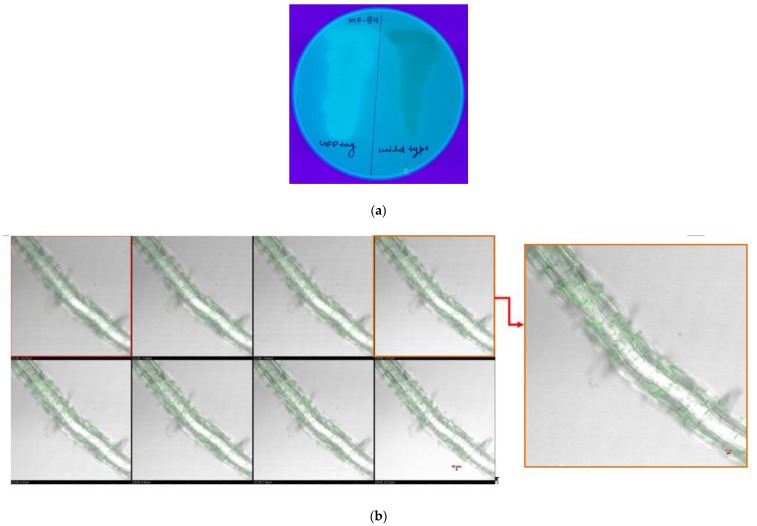
(**a**)GFP tagging of *Pseudomonas geniculata* MF-84; (**b**)Visualization of GFP tagged *Pseudomonas geniculata* MF-84 in the maize roots through Confocal Scanning Laser Microscope (10×).

However, initial confocal microphotographs indicated that *P. geniculata* MF-84 colonize the internal root tissues as endophytes. To investigate the endophytic nature of *P. geniculata* MF-84, GFP tagging was done. A number of GFP-tagged trans-conjugants were selected and plated separately on LB media. These trans-conjugants flourished under UV light (Figure 3a). Further, treated roots indicated that GFP tagged *P. geniculata* MF-84 enters inside the roots (Figure 3b).

**Figure 4:**
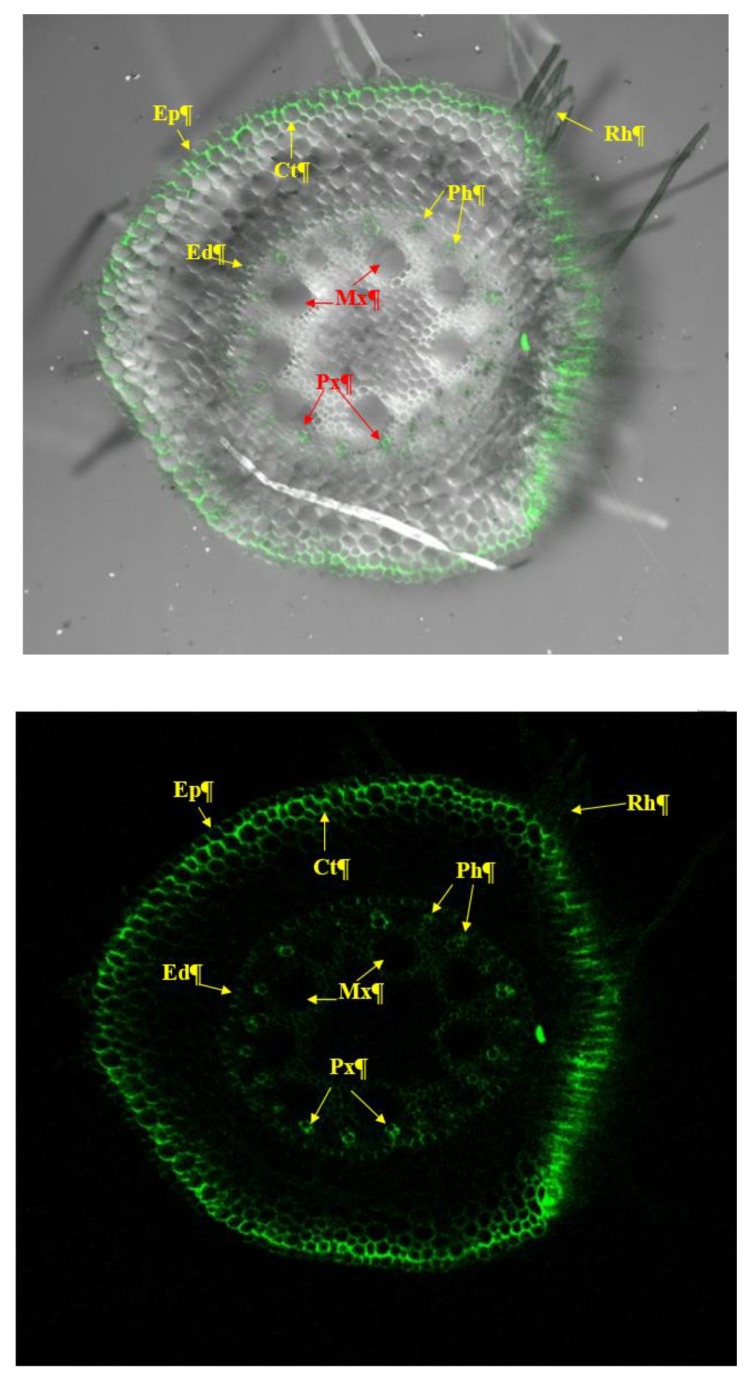
Localization of GFP-tagged *Pseudomonas* *geniculata* MF-84 in maize root tissues (endophyticnature) under a confocal scanning laser microscope (10×).

Bacterial colonization was visualized in the epidermis, cortical tissues, endodermis and vascular bundles including proto-xylem, meta-xylem, phloem and bundle sheath (Figure 4). Confocal microscopic examination clearly showed that entire root was showing bacterial signal as green colour at 15 DAS.

### 3.3. Effects of Seed-Biopriming on Biochemical Properties of Maize

**Figure 5:**
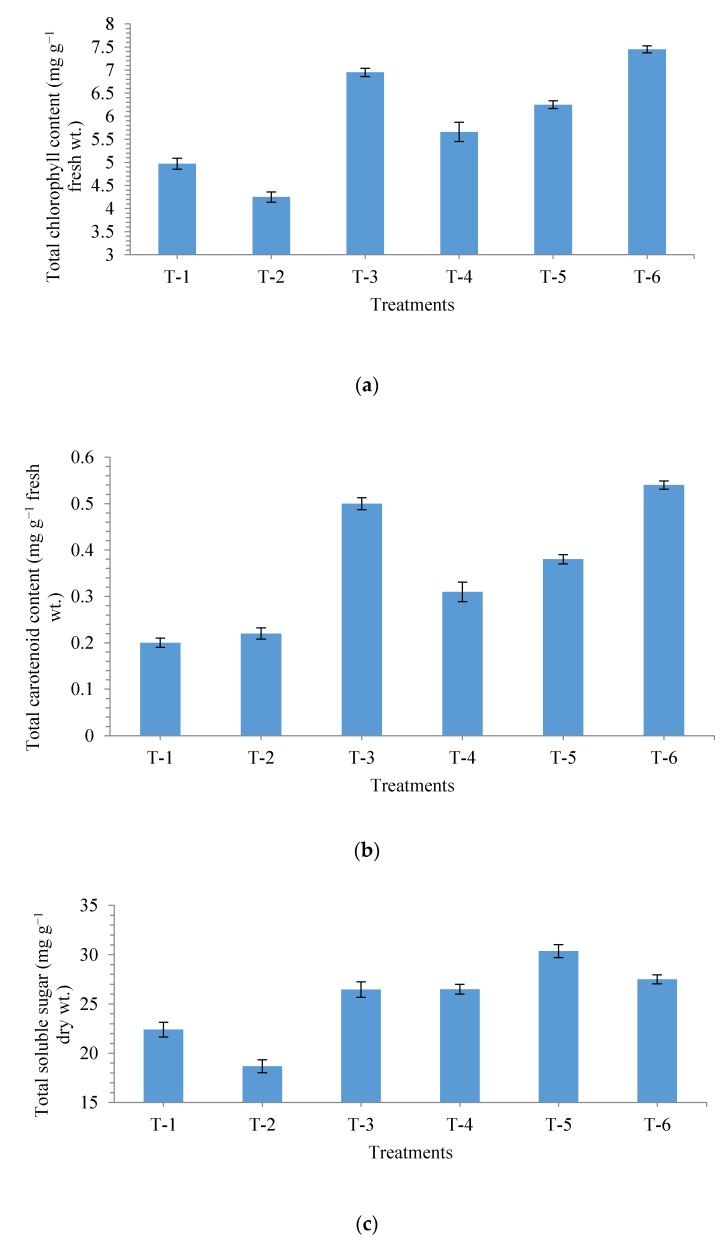

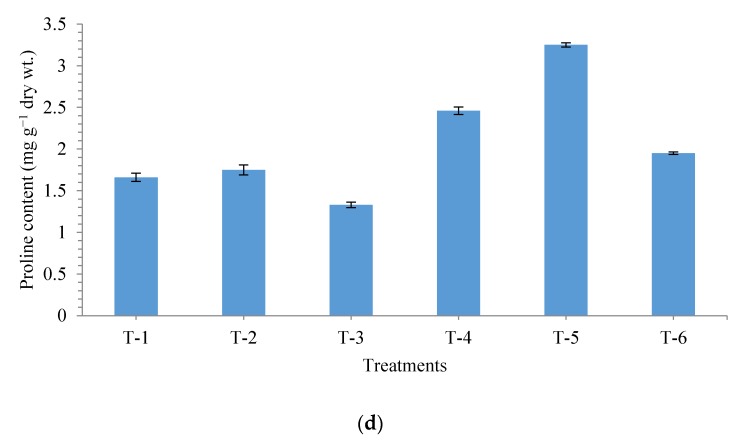
Effects of seed bio-primingon (**a**) total chlorophyll content, (**b**) total carotenoids content, (**c**) total soluble sugar, and (**d**) proline content in maize at 30 days after sowing. Treatments were: T_1_-Saline sodic soil (Negative control), T_2_-Non-saline sodic soil + Salt (NaCl 150 mM), T_3_-Non-saline sodic soil (Positive control), T_4_-Saline sodic soil + *Pseudomonas geniculata* MF-84, T_5_-Non-saline sodic soil + Salt (NaCl 150 mM) + *Pseudomonas geniculata* MF-84 and T_6_-Non-saline sodic soil + *Pseudomonas geniculata* MF-84. Data are mean ± SEM (*n* = 5).

Application of *P. geniculata* MF-84 modulated physiological and biochemical properties of maize grown in salt stressed soil. The activation and accumulation of total chlorophyll, carotenoids, total soluble sugar and proline were studied in the *P. geniculata* MF-84 bio-primed maize grown under salt stress conditions. Maximum chlorophyll content was recorded in the maize plants grown in non-saline soil and bio-primed with *P. geniculata* MF-84 (7.45 mg g^−1^ fresh wt.) followed by those in non−saline soil (6.95 mg g^−1^ fresh wt.) and non-saline soil with salt (NaCl 150 mM) and bio-primed with strain MF-84 (6.25 mg g^−1^ fresh wt.) at 30 DAS in nethouse experiments (Figure 5a). However, amount of chlorophyll was recorded least in the plants grown in non-saline soil with NaCl 150 mM (4.25 mg g^−1^ fresh wt.) and saline sodic soil (4.97 mg g^−1^ fresh wt.). The enhanced chlorophyll content was also discernible from increased plant growth parameters such as shoot and root length, fresh and dry biomass in the in the plants with and without the test strain MF-84 compared to untreated plants under salt stressed and non-stressed conditions (Figure 5a).

Plants bio-primed with MF-84 promoted accumulation of total carotenoids in the leaves significantly (*p* < 0.05) under salt stressed and non-stressed conditions (Figure 5b). Similar to chlorophyll content, maximum carotenoids content was also recorded in the plant grown in non-saline soil and treated with bacterial strain MF-84 (0.54 mg g^−1^ fresh wt.) followed by non-saline soil (0.50 mg g^−1^ fresh wt.). Other treatments showed more or less similar trends as recorded for chlorophyll content (Figure 5b).

Plants subjected to salt stress tend to over produce total soluble sugar and prolinein plant tissues. Quantitative profile of total soluble sugar and prolinein maize plants varied significantly between the plants bio-primed with *P. geniculata* MF-84 under salt stressed and non-stressed conditions. The highest content of soluble sugar and proline (30.36 and 3.25 mg g^−1^ dry wt., respectively) was recorded in plants bio-primed with *P. geniculata* strain MF-84 and sown in pots containing non-saline soil with salt (NaCl 150 mM) followed by those grown in saline sodic soil and bio-primed with strain MF-84. However, least amount of soluble sugar and proline was recorded in the plants sown in non-saline soil along with salt (T_-2_) and non-saline soil (T_-3_), respectively, compared to other treatments (Figure 5c,d).

### 3.4. Effects of Seed Bio-Priming on Antioxidant Enzymes in Maize

**Figure 6:**
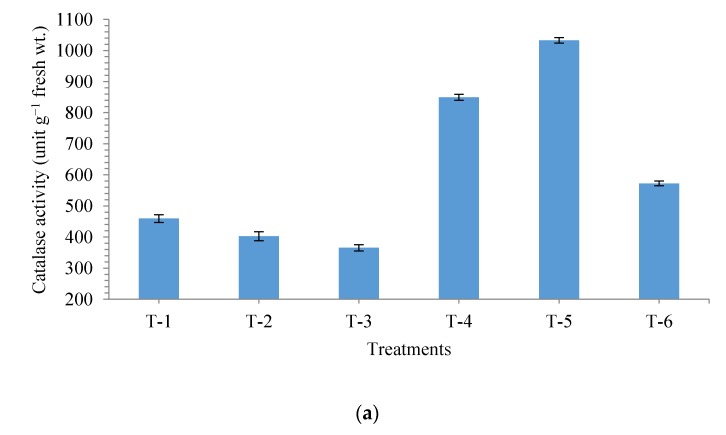

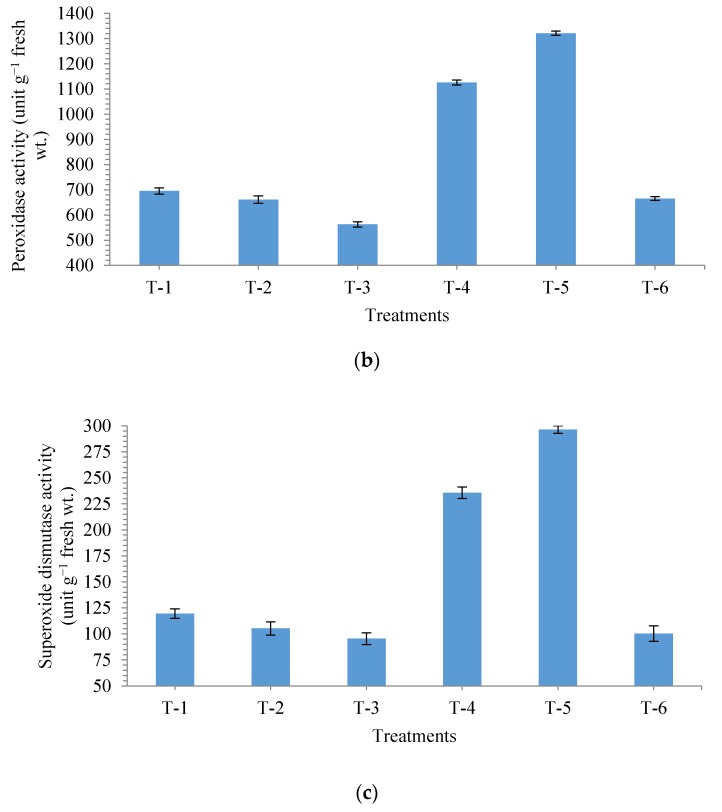
Effects of seed bio-primingon activity of antioxidant enzymes (**a**) catalase activity, (**b**) peroxidase activity, and (**c**) superoxide dismutase activity in maize at 30 days after sowing. Treatments were: T_1_-Saline sodic soil (Negative control), T_2_-Non-saline sodic soil + Salt (NaCl 150 mM), T_3_-Non-saline sodic soil (Positive control), T_4_-Saline sodic soil + *Pseudomonas geniculata* MF-84, T_5_-Non-saline sodic soil + Salt (NaCl 150 mM) + *Pseudomonas geniculata* MF-84 and T_6_-Non-saline sodic soil + *Pseudomonas geniculata* MF-84. Data are mean ± SEM (*n* = 5).

In general, plants tend to overproduce antioxidant enzymes under salt stress conditions. In the present study, effect of seed bio-priming on catalase (CAT), peroxidase (POx) and superoxide dismutase (SOD) activity was studied in the maize plants grown in salt stressed and non-stressed soil. Surprisingly, these enzymes were expressed in a similar way across the treatments. The activity of CAT, POx and SOD increased significantly in the plant roots bio-primed with strain MF-84 and sown in non-saline soil containing salt (NaCl 150 mM) (T_-5_) followed by those bio-primed with strain MF-84 and grown in saline sodic soil (T_-4_) (Figure 6a–c, respectively). However, plant grown in non-saline soil without bacterial treatment expressed low enzymatic activities in their roots compared to other treatments (Figure 6).

### 3.5. Effects of Seed Bio-Priming on Plant Growth

Maize plants were harvested at 30 DAS and plant growth parameters were measured and compared with positive control (T_-3_) as seen in Table 3. The data indicate that the positive impact of *P. geniculata* strain MF-84 on the plant growth and growth attributes under salt stress and non-stress conditions was obvious. The highest shoot length (28.35 cm), root length (24.78 cm), fresh wt. of shoot (8.05 g), fresh wt. of root (5.25 g) and dry wt. of shoot (2.25 g) and root (1.67 g) were recorded in the plants bio-primed with *P. geniculata* strain MF-84 grown in non-saline sodic soil (T_-6_) at 30 DAS. A closer look on the plant root and shoot system reveals that the root and shoot biomass was significantly higher in positive control (T_-3_). However, enhanced root and shoot biomass was recorded in plants treated with *P. geniculata* strain MF-84 grown in non-saline sodic soil at 30 DAS (T_-6_), indicative of the establishment of symbiotic mutual relationships between the *P. geniculata* strain MF-84 and the plant roots under salt stressed and non-stressed conditions. In fact; without bio-inoculant, all plants exhibited reduction in plant growth and biomass accumulation under salt stressed and non-stressed conditions (Table 3).

### 3.6. Effects of Seed Bio-Priming on Uptake of Na^+^, K^+^ and Ca^2+^

**Figure 7:**
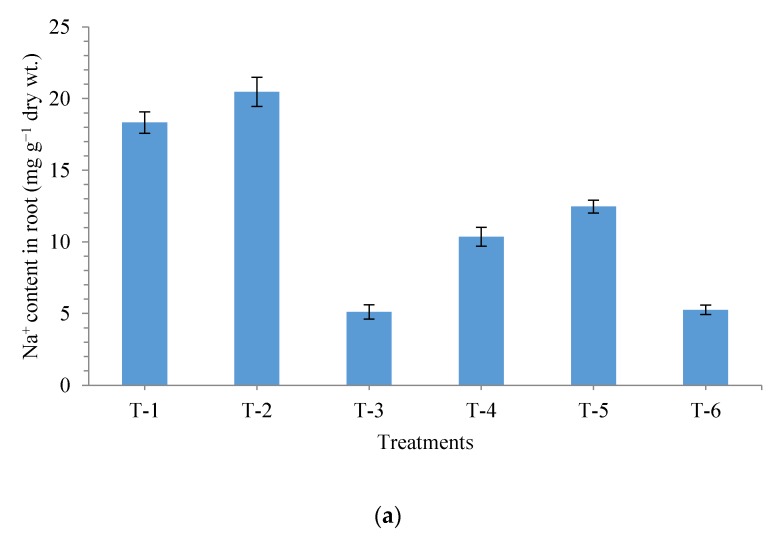

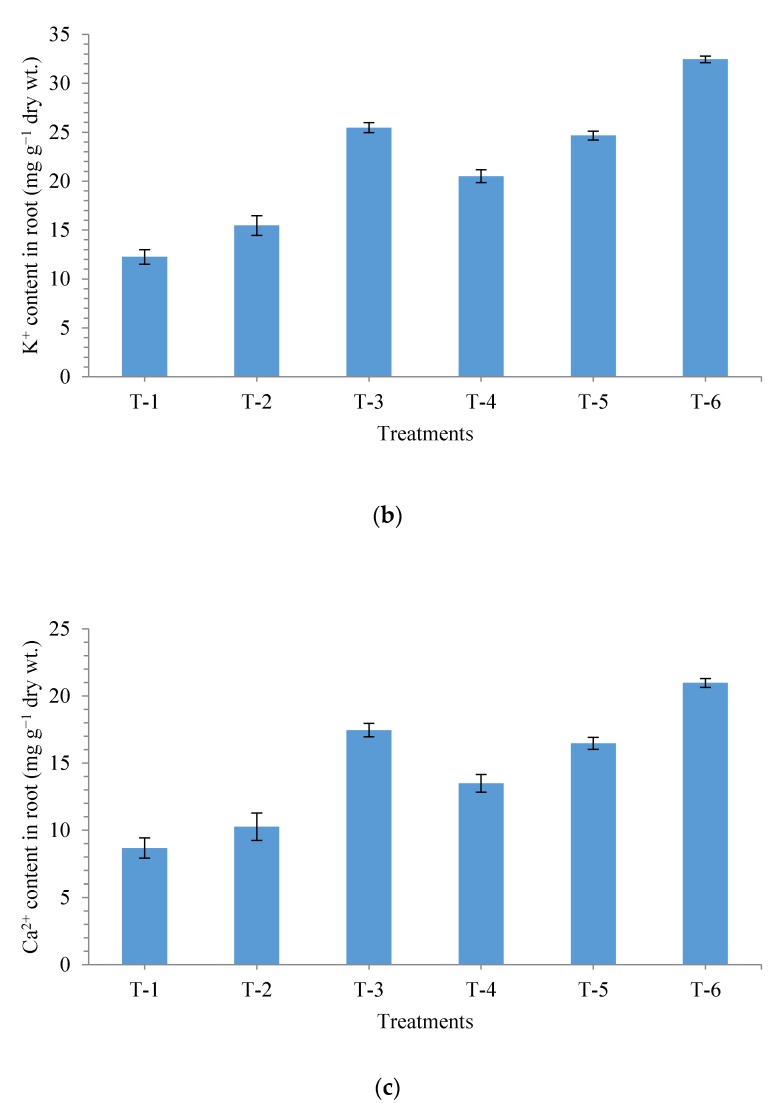
Effects of seed bio-priming on uptake of cations (**a**) Na^+^ content in roots, (**b**) K^+^ content in roots, and (**c**) Ca^2+^ content in roots at 30 days after sowing. Treatments were: T_1_-Saline sodic soil (Negative control), T_2_-Non-saline sodic soil + Salt (NaCl 150 mM), T_3_-Non-saline sodic soil (Positive control), T_4_-Saline sodic soil + *Pseudomonas geniculata* MF-84, T_5_-Non-saline sodic soil + Salt (NaCl 150 mM) + *Pseudomonas geniculata* MF-84 and T_6_-Non-saline sodic soil + *Pseudomonas geniculata* MF-84. Data are mean ± SEM (*n* = 5).

For better understanding of impact of *P. geniculata* strain MF-84 on uptake of Na^+^, K^+^ and Ca^2+^, the content of Na^+^, K^+^ and Ca^2+^ was measured in plant roots at 30 DAS. Significantly higher amount of Na^+^ was recorded in the roots of plants grown in non-saline soil with salt (20.47 mg g^−1^ dry wt.) followed by that of plants grown in saline sodic soil (18.33 mg g^−1^ dry wt.). However, contrary to Na^+^ in the same treatments, least amounts of K^+^ and Ca^2+^ were recorded (Figure 7a–c).

Application of *P. geniculate* MF-84 significantly reduces the uptake of Na^+^ (Figure 7a) and increase uptake of K^+^ (Figure 7b) and Ca^2+^ (Figure 7c) across the treatments and conditions. The least Na^+^ uptake was recorded in the plants grown in non-saline soil (5.10 mg g^−1^ dry wt.) followed by plants bio-primed with strain MF-84 and grown in non-saline soil (5.25 mg g^−1^ dry wt.). However, maximum K^+^ (32.46 mg g^−1^ dry wt.) and Ca^2+^ (20.97 mg g^−1^ dry wt.) was recorded in the plants bio-primed with strain MF-84 and grown in non-saline soil followed by those grown in non-saline soil (Figure 7b,c).

## 4. Discussion

The present study demonstrates the impact of seed bio-priming with salt-tolerant endophytic *P. Geniculata* which modulates the biochemical responses and provides ecological fitness to the maize (*Zea mays* L.) plants grown in saline sodic soil. *P. geniculate* MF-84 isolated from maize rhizosphere exhibited better salt tolerance and has plant growth promoting attributes as indicated by results of the present study. This is in agreement with the Gopalakrishnan et al. who demonstrated that *P. geniculata* IC-76 isolate produces indole acetic acid, siderophore, hydrocyanic acid, cellulase, protease, and β-1,3-glucanase [36]. Confocal microscopic study revealed that GFP tagged *P. geniculate* MF-84 colonized maize root vigorously and reached up to vascular bundles in roots. GFP tagging has been commonly used to determine spatial and temporal movement of an organism in the host system and is being exploited to detect the localization of many plant-associated bacteria [37]. GFP-tagging provides a most useful tool to localized individual cells or clusters of bacterial cell during colonization over a period of time in living tissues and even after cell death [38]. The role of microorganisms in the management of biotic and abiotic stresses is gaining importance and PGPR elicited tolerance to abiotic stresses has been studied and reviewed by various workers [1,39,40]. Salt tolerant PGP microbes enhance host plant growth by exploiting different mechanisms under salt stressed conditions. In general, plant growth promoting rhizobacteria and plant growth promoting endophytic bacteria produce IAA which is known to stimulate seed germination, initiate lateral root formation and thereby increasing root surface area facilitating greater access to water and soil nutrients by the host plant [9,13,41,42]. In the present study, *P. geniculate* MF-84 was found to produce IAA in the media and to increase root biomass directly and/or indirectly under salt stress. 

Further, the microorganisms isolated from salt stressed habitats possess stress tolerance ability along with the PGP traits. Salt-tolerant microbes can impart high degree of salt tolerance to the plants grown in salt affected soils [1,43,44,45]. Application of salt tolerant microorganisms alleviates negative effects of salt stress in wide range of crop plants and thus opening a new and emerging area where microorganisms can be exploited [3,15,46]. Microorganisms elicit a number of pathways/cascades upon interaction with their host plants over a period of time. Some of them are directly involved in the alleviation of salt stress, while others play indirect role in keeping plant more robust and healthy even under stress [22,26,47,48,49]. A number of bacteria produce exo-polysaccharides and plants treated with EPS producing bacteria exhibited higher tolerance to salinity stress as they improve soil structure by re-structuring soil aggregates [1,29]. Furthermore, EPS bind to cations including Na^+^ on their surface and thereby making them unavailable to the plants under saline/salt stress [1,29]. Higher concentrations of Na^+^ in root cells cause osmotic burst leading to electrolyte leakage [1,50]. Present study revealed that plants treated with *P. geniculate* MF-84 enhanced synthesis and accumulation of proline and soluble sugars in the plant system. These biomolecules play an important role in membrane stability and regulate osmotic burst in root cells of plants under salt stress [1,50]. Similarly, Chen et al. reported the role of proline and organic solutes accumulation in the drought and salt tolerance in plants [43]. Bano and Fatima reported that increased level of proline decreases electrolyte leakage, maintains relative water content and increases uptake of K^+^ and Ca^2+^ ions providing high salt tolerance in maize plants inoculated with *Pseudomonas* spp. [23]. It was also shown that plants treated with *P. geniculata* MF-84 over produce antioxidant enzymes like CAT, Pox and SOD in maize root. Paul and Lade reported that activities of antioxidant enzymes generally increase when plants are subjected to biotic or abiotic stress [24]. Superoxide dismutase and peroxidase are generally involved in eliminating reactive oxygen species [25,44,50], reducing lipid peroxidation [44] and in increasing membrane thermostability [24]. Excess sodium and more importantly, chloride in plant cells has the potential to affect plant enzymes and cause cell swelling resulting in reduced energy production and other physiological changes [1,49]. Moreover, *P. geniculate* MF-84 restricts uptake of Na^+^ and increases uptake of K^+^ and Ca^2+^ across the treatments of salt stress. Na^+^-K^+^ exchange pump on the plasma membrane or vacuole plays a key role in regulatory mechanisms in maintaining ion homeostasis in the cell [1,51,52,53,54]. Endophytic/rhizospheric microbes elicit cellular signaling which up-regulates biochemical pathways related to salt stress and protect plants from negative effects of salt [55,56,57,58,59]. An increase in the uptake of Na^+^ or decrease in the uptake of Ca^2+^ and K^+^ in the roots leads to nutritional imbalances. Accumulation of excessive Na^+^ may cause metabolic disturbances in processes where low Na^+^ and high K^+^ or Ca^2+^ are required for optimum function [46,48,60,61]. Results indicated that plants bio-primed with *P. geniculata* MF-84 showed better shoot and root length, and plant biomass under salt stress. Endophytic and rhizospheric microorganisms mineralize nutrients in the soil and make them available to plants. Further, many microbes have been found which directly synthesize growth hormones or elicit plant system to produce and regulate essential growth regulators in an indirect way finally resulting in better plant growth [62,63,64]. However, further evidences are needed to verify the exact role of bioinoculant along with other putative mechanisms participating in microbe-mediated salinity tolerance in maize crop [65,66,67,68].

## 5. Conclusions

The salt tolerant endophytic *P. geniculata* MF-84 is a well-established microorganism having good plant growth promoting traits. SEM and confocal microscopy studies clearly indicated that the test strain has root colonization potential and can colonize more than 70% of roots within a short period of 15 days. Application of *P. geniculata* MF-84 increased chlorophyll and carotenoids content significantly even under salt stress condition and gave better support to maize plants for their establishment. Further, it was found to increase proline content and soluble sugar which maintain turgor pressure in root cell in the plants. A significant increase in antioxidant enzymes was recorded in the plant treated with *P. geniculata* MF-84 protecting plant cells from toxic effects of reactive oxygen species. *P. geniculata* MF-84 modulated the uptake of Na^+^, K^+^, and Ca^2+^ leading to better plant growth under salt stress. Thus, results of the present study are sufficient to prove *P. geniculata* MF-84 an excellent plant growth promoting agent under saline and non-saline conditions.

## Figures and Tables

**Table 1:** Initial physico-biochemical properties of experimental soil.

Sample No.	Soil Properties	Saline Sodic Soil	Non-Saline Sodic Soil
1.	Soil type	Silty clay loam	Silty clay loam
2.	pH	8.5	7.2
3.	EC (dS m^−1^)	2.58	0.45
4.	OC (g kg^−1^)	3.50	5.50
5.	OM (g kg^−1^)	7.25	9.46
6.	Available macronutrients (kg ha^−1^)		
I.	Nitrogen	175.33	205.66
II.	Phosphorous	32.45	42.36
III.	Potassium	142.50	162.75
IV.	Sulphur	6.45	8.26
V.			
7.	Available micronutrients (mg kg^−1^)		
I.	Fe	4.26	11.50
II.	Cu	0.52	0.91
III.	Zn	0.75	0.82
IV.	B	0.09	0.12

**Table 2:** Treatments code and details.

Treatments Code	Treatment Details
T_1_	Saline sodic soil (Negative control)
T_2_	Non-saline sodic soil + Salt (NaCl 150 mM)
T_3_	Non-saline sodic soil (Positive control)
T_4_	Saline sodic soil + *Pseudomonas geniculata* MF-84
T_5_	Non-saline sodic soil + Salt (NaCl 150 mM) + *Pseudomonas geniculata* MF-84
T_6_	Non-saline sodic soil + *Pseudomonas geniculata* MF-84

**Table 3:** Effect of seed biopriming with *Pseudomonas geniculata* MF-84 on plant growth attributes in maize at 30 days of sowing under nethouse conditions.

Treatments	Shoot Length (cm)	Root Length (cm)	Fresh wt. of Shoot (g)	Fresh wt. of Root (g)	Dry wt. of Shoot (g)	Dry wt. of Root (g)
T_1_	15.25 ± 0.75 ^d^	12.50 ± 0.66 ^c^	4.97 ± 0.22 ^d^	2.15 ± 0.16 ^e^	1.05 ± 0.05 ^d^	0.66 ± 0.01 ^c^
T_2_	14.37 ± 1.05 ^d^	10.47 ± 0.54 ^d^	3.42 ± 0.35 ^e^	2.05 ± 0.25 ^e^	0.97 ± 0.12 ^d^	0.75 ± 0.03 ^c^
T_3_	22.75 ± 1.33 ^c^	18.36 ± 0.85 ^b^	6.50 ± 0.25 ^b^	3.97 ± 0.31 ^b^	1.66 ± 0.09 ^b^	1.02 ± 0.04 ^b^
T_4_	21.92 ± 0.95 ^c^	17.89 ± 1.25 ^b^	5.90 ± 0.15 ^c^	3.15 ± 0.17 ^d^	1.35 ± 0.04 ^c^	1.15 ± 0.04 ^b^
T_5_	23.46 ± 1.15 ^b^	20.50 ± 1.01 ^b^	4.87 ± 0.25 ^d^	3.36 ± 0.21 ^c^	1.42 ± 0.05 ^c^	1.25 ± 0.05 ^b^
T_6_	28.35 ± 1.26 ^a^	24.78 ± 0.96 ^a^	8.05 ± 0.33 ^a^	5.25 ± 0.28 ^a^	2.25 ± 0.11 ^a^	1.76 ± 0.02 ^a^

T_1_-Saline sodic soil (Negative control), T_2_-Non-saline sodic soil + Salt (NaCl 150 mM), T_3_-Non-saline sodic soil (Positive control), T_4_-Saline sodic soil + *Pseudomonas geniculata* MF-84, T_5_-Non-saline sodic soil + Salt (NaCl 150 mM) + *Pseudomonas geniculata* MF-84 andT_6_-Non-saline sodic soil + *Pseudomonas geniculata* MF-84. Data are mean ± SEM (*n* = 5), data with different letters show significant difference in column data in randomized block design test at *p* < 0.05 under Duncan’s multiple range test.
